# Accurately estimating correlations between demographic parameters: A comment on Deane et al. (2023)

**DOI:** 10.1002/ece3.70286

**Published:** 2024-09-17

**Authors:** Thomas V. Riecke, Dan Gibson, James S. Sedinger

**Affiliations:** ^1^ Wildlife Biology Program University of Montana Missoula Montana USA; ^2^ Department of Fisheries, Wildlife and Conservation Biology University of Minnesota St. Paul Minnesota USA; ^3^ Department of Natural Resources and Environmental Science University of Nevada Reno Nevada USA

**Keywords:** band‐recovery models, Bayesian, demography, harvest, hunting, multivariate normal, prior

## Abstract

Estimating correlations among demographic parameters is an important method in population ecology. A recent paper by Deane et al. (*Ecology and Evolution* 13:e9847, 2023) attempted to explore the effects of different priors for covariance matrices on inference when using mark‐recovery data. Unfortunately, Deane et al. (2023) made a mistake when parameterizing some of their models. Rather than exploring the effects of different priors, they examined the effects of the use of incorrect equations on inference. In this manuscript, we clearly describe the mistake in Deane et al. (2023). We then demonstrate the use of an alternative and appropriate method and reach different conclusions regarding the effects of priors on inference. Consistent with other recent literature, informative inverse Wishart priors can lead to flawed inference, while vague priors on covariance matrix components have little impact when sample sizes are adequate.

## Graphical abstract

Deane et al. (*Ecology and Evolution* 13:e9847, 2023) attempted to explore the effects of prior choice on inference for band‐recovery models. In this response we highlight alternative and accurate approaches, and reexamine their conclusions in light of the use of correct equations.

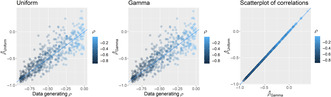

## 1 INTRODUCTION

Estimating temporal or within‐individual correlations among demographic parameters from capture‐reencounter data is often employed to investigate community (Zipkin et al., 2023), population (Riecke et al., 2019), and evolutionary (Fay et al., 2022) demography. Models that estimate correlated random effects from multivariate normal distributions can be useful for these types of analyses and allow for inference regarding processes such as synchronous responses to environmental stressors (Riecke et al., 2019), the effects of human harvest on survival rates of wildlife populations (Ergon et al., 2018), or latent variation in individual quality (Fay et al., 2022). When these analyses are performed using Bayesian statistical approaches, prior choice can affect posterior estimates and subsequent inference about the strength of correlations and resulting parameter estimates. Recent papers have sought to explore the consequences of prior choice on estimates of correlations at both the population‐ and individual‐level using capture‐recapture data (Fay et al., 2022; Riecke et al., 2019), and concluded that commonly used priors for covariance matrices, such as the conjugate inverse‐Wishart distribution, may lead to erroneous inference.

Deane et al. (2023) explored a similar line of inquiry using mark‐recovery data. Specifically, they attempted to examine the effects of three prior structures for covariance matrices, as well as different sample sizes (i.e., number of releases), on posterior estimates of the correlation between survival and band‐recovery rates. This approach may allow for inference regarding the effects of hunting on survival in harvested populations. Two of the three priors (‘Wishart’ and ‘Uniform’) used in Deane et al. for capture‐recovery data are identical to priors described in Riecke et al. (2019) for capture‐recapture data. Deane et al. (2023) also described a third prior, which they referred to as the ‘Gamma’ prior. Unfortunately, incorrect equations related to the ‘Gamma’ prior were used in both the text and code of Deane et al. (2023) that led to uninterpretable parameter estimates and associated erroneous inference.

In this Letter to the Editor, we first demonstrate basic errors in equations in Deane et al. (2023) so that others can avoid similar mistakes (Section 2). We then demonstrate an accurate and effective method for placing gamma priors on the standard deviations of a covariance matrix for bivariate normal distributions (Section 3). Third, we discuss some of the errors in interpretation in Deane et al. (2023) after obtaining parameter estimates from correctly parameterized models (Section 4). Finally, we note that many different effective approaches for placing priors on covariance matrices exist, highlight several key areas of agreement with Deane et al. (2023), and describe best practices for avoiding similar errors in the future (Section 5).

## 2 ERRORS IN IMPLEMENTATION

Deane et al. (2023) estimated correlations (*ρ*) between logit‐transformed survival (*S*) and band‐recovery (*f*) probabilities. Given a correctly specified covariance matrix (**Σ**) equal to,

(1) $$\Sigma =\left[\begin{array}{cc} \sigma_{S}^{2} & \sigma_{S}\sigma_{f}\rho \\ \sigma_{S}\sigma_{f}\rho & \sigma_{f}^{2} \end{array}\right],$$

the correct formulation of a precision matrix (i.e., the inverse of a covariance matrix) is,

(2) $$\Sigma^{-1}=\left[\begin{array}{cc} \frac{\sigma_{f}^{2}}{\sigma_{S}^{2}\sigma_{f}^{2}\left(1-\rho^{2}\right)} & \frac{-\sigma_{S}\sigma_{f}\rho }{\sigma_{S}^{2}\sigma_{f}^{2}\left(1-\rho^{2}\right)}\\ \frac{-\sigma_{S}\sigma_{f}\rho }{\sigma_{S}^{2}\sigma_{f}^{2}\left(1-\rho^{2}\right)} & \frac{\sigma_{S}^{2}}{\sigma_{S}^{2}\sigma_{f}^{2}\left(1-\rho^{2}\right)} \end{array}\right].$$

Precision matrices are useful for the calculation of partial correlations, and are used by some MCMC samplers (e.g., JAGS; Plummer, 2003) to parameterize multivariate normal distributions. In equation (7) of their manuscript, Deane et al. (2023) specified the inverse of a covariance matrix (i.e., the precision matrix) as,

(3) $$\Sigma^{-1}=\left[\begin{array}{cc} \frac{1}{\sigma_{S}^{2}} & \left(\frac{1}{\sigma_{S}\sigma_{f}}\right)\rho^{*}\\ \left(\frac{1}{\sigma_{S}\sigma_{f}}\right)\rho^{*} & \frac{1}{\sigma_{f}^{2}} \end{array}\right].$$

This differs from Equation (2) and is incorrect (Lauritzen, 1996). The Authors indicated in the text that this parameterization (equation 7 in Deane et al., 2023) is ‘like the default priors specified for multivariate normal distributions in Program MARK (White & Burnham, 1999)’. This statement is inaccurate. The gamma priors used in Program MARK are assigned to standard deviations, not precisions. More importantly, the equations used by Program MARK to specify covariance matrices are valid (G. C. White, personal communication).

Deane et al. (2023) used a second incorrect formulation of this equation in the code they wrote to analyze their simulated data as well as the real mallard data, where they formulated the covariance (**Σ**) and precision (**Ω**) matrices as,

(4) $$\begin{array}{c} \Sigma =\Omega^{-1},\\ \Omega =\left[\begin{array}{cc} \omega_{11} & \left(\sqrt{\omega_{11}\omega_{22}}\right)\rho^{*}\\ \left(\sqrt{\omega_{11}\omega_{22}}\right)\rho^{*} & \omega_{22} \end{array}\right],\\ \begin{array}{c} \begin{array}{c} \omega_{11}\sim \text{gamma}\left(1.001,0.001\right),\\ \omega_{22}\sim \text{gamma}\left(1.001,0.001\right), \end{array}\\ \rho^{*}=\left(2\times \rho_{\text{prior}}^{*}\right)-1\\ \rho_{\text{prior}}^{*}\sim \text{beta}\left(1,1\right) \end{array} \end{array}$$

This is also incorrect (Lauritzen, 1996). To be clear, Deane et al. (2023) assumed that they were placing a gamma prior on the precisions $\left(\tau =\frac{1}{\sigma^{2}}\right)$ of survival and band‐recovery probabilities. In both the text and the code, they assigned this prior to a complex function including all the parameters in the covariance matrix. For example, the authors assign this prior to $\Omega_{11}=\frac{\sigma_{f}^{2}}{\sigma_{S}^{2}\sigma_{f}^{2}\left(1-\rho^{2}\right)}$,

(5) $$\Omega_{11}\sim \text{gamma}\left(1.001,0.001\right).$$

Unfortunately, this parameterization is incorrect. The disparity between the correct formulation of a precision matrix and the two incorrect equations used by Deane et al. (2023) led to errors in the results of their manuscript. Furthermore, it led to an erroneous conclusion that the use of gamma priors for precisions resulted in estimates of correlations that are more extreme (i.e., further from 0) than truth. In Section 3, we demonstrate that this conclusion was inaccurate. Unfortunately, given the use of incorrect equations in their analyses, we suggest that the estimates and conclusions from Deane et al. (2023) regarding the ‘Gamma’ prior they described are unreliable. In the next section of this letter, we demonstrate how to appropriately apply gamma priors to standard deviations of a covariance matrix.

## 3 CORRECTLY IMPLEMENTING A GAMMA PRIOR

### 3.1 Data simulation and analysis

Here we demonstrate how to specify gamma priors for the standard deviations of demographic parameters estimated using a bivariate normal distribution. For each simulation (*n* = 500) we generated mean hunting $\left(\mu_{\kappa }=-1\right)$ and natural $\left(\mu_{\eta }=-1\right)$ mortality hazard rates. We then simulated correlated temporal variation $\left(\varepsilon \right)$ between the two mortality hazard rates given a multivariate normal distribution with mean zero and an appropriate covariance matrix constructed using values of **σ** and *ρ* that varied among simulations,

(6) $$\begin{array}{c} \sigma \sim \text{gamma}\left(20,80\right),\\ \rho \sim \text{uniform}\left(-0.9,0\right),\\ \begin{array}{c} \Sigma =\left[\begin{array}{cc} \sigma_{\kappa }^{2} & \sigma_{\kappa }\sigma_{\eta }\rho \\ \sigma_{\kappa }\sigma_{\eta }\rho & \sigma_{\eta }^{2} \end{array}\right],\\ \varepsilon \sim \text{bivariate normal}\left(0,\Sigma \right). \end{array} \end{array}$$

We simulated correlated hunting $\left(h_{\kappa }\right)$ and natural $\left(h_{\eta }\right)$ mortality hazard rates (Ergon et al., 2018) and transformed those rates to survival (*S*), natural mortality (*η*), hunting mortality (*κ*) and band‐recovery (*f*) probabilities assuming constant crippling loss (*c* = .2) and band reporting (*b* = .5) probabilities to correspond to classic ring‐recovery models (Brownie et al., 1985),

(7) $$\begin{array}{c} \ln\left(h_{\kappa ,t}\right)=\mu_{\kappa }+\varepsilon_{\kappa ,t}\\ \ln\left(h_{\eta ,t}\right)=\mu_{\eta }+\varepsilon_{\eta ,t}\\ \begin{array}{c} S_{t}=e^{-\left(h_{\kappa ,t}+h_{\eta ,t}\right)}\\ \eta_{t}=\left(1-S_{t}\right)\frac{h_{\eta ,t}}{h_{\eta ,t}+h_{\kappa ,t}}\\ \begin{array}{c} \kappa_{t}=\left(1-S_{t}\right)\frac{h_{\kappa ,t}}{h_{\eta ,t}+h_{\kappa ,t}}\\ f_{t}=\kappa_{t}\left(1-c\right)b \end{array} \end{array} \end{array}$$

We then simulated mark‐recovery *m*‐arrays (*M*), where we first specified cell probabilities (*P*) for each cell of the m‐array, and then simulated outcomes (i.e., band recoveries) as a multinomial trial,

(8) $$\begin{array}{c} P_{\mathrm{ij}}=\left\{\begin{array}{cc} 0 & i>j\\ f_{i} & i=j\\ \left(\prod_{k=i}^{k=j-1}S_{k}\right)f_{j} & i<j<T+1\\ 1-\sum_{k=1}^{k=T}P_{\mathrm{ik}} & j=T+1 \end{array}\;\right.\\ M_{t,1:\left(T+1\right)}\sim \text{multinomial}\left(10,000,P_{t,1:\left(T+1\right)}\right). \end{array}$$

Like Deane et al.'s (2023) ‘intensive’ monitoring scenario, we released 10k individuals per year for 36 years. We analyzed each simulated dataset (Supplementary Code) using the data generating model and two different (‘Uniform’ and ‘Gamma’) priors on the standard deviations to recover parameter estimates from the simulated data. In the first model, which follows the ‘Uniform’ parameterization described in Riecke et al. (2019), we applied vague uniform priors to the standard deviations of the mortality hazard rates, $\sigma \sim \text{uniform}\left(0,5\right)$. In the second model, we applied vague gamma priors to the standard deviations of the mortality hazard rates, $\sigma \sim \text{gamma}\left(1,1\right)$. We specified a uniform prior for the correlation between mortality hazard rates, $\rho \sim \text{uniform}\left(-1,1\right)$ and weakly informative priors for the means of the mortality hazard rates, $\mu \sim \text{normal}\left(-1,1\right)$ for both the ‘Uniform’ and ‘Gamma’ approaches. We fixed the values of crippling loss probability, c=.2, and band reporting probability, b=.5, for both models. Readers should note that our simulation study differs slightly from Deane et al. (2023) in that we did not explore the effects of different numbers of releases on inference. Furthermore, we use Stan (Stan Development Team, 2024b) rather than JAGS to take advantage of more efficient sampling. Thus, the goal of our simulation was simply to demonstrate that vague gamma and uniform priors on the standard deviations of mortality hazard rates or probabilities should not have major impacts on inference if researchers use appropriate equations.

### 3.2 Computational details

We conducted all analyses in R (R Core Team, 2023) and Stan (Stan Development Team, 2024a) using the rstan package (Stan Development Team, 2024b). For each simulation (*n* = 500) we sampled four MCMC chains for 25k iterations, discarding the first 10k iterations and retaining every 5th sample. In the following figures we report posterior distribution medians from each iteration. We excluded simulations (*n* = 0) in which either model type did not achieve >1000 effective samples or $\hat{R}<1.01$ for the correlation (*ρ*) parameters. This led to a total of 500 simulations that were used to calculate summary statistics.

### 3.3 Results

**FIGURE 1:**
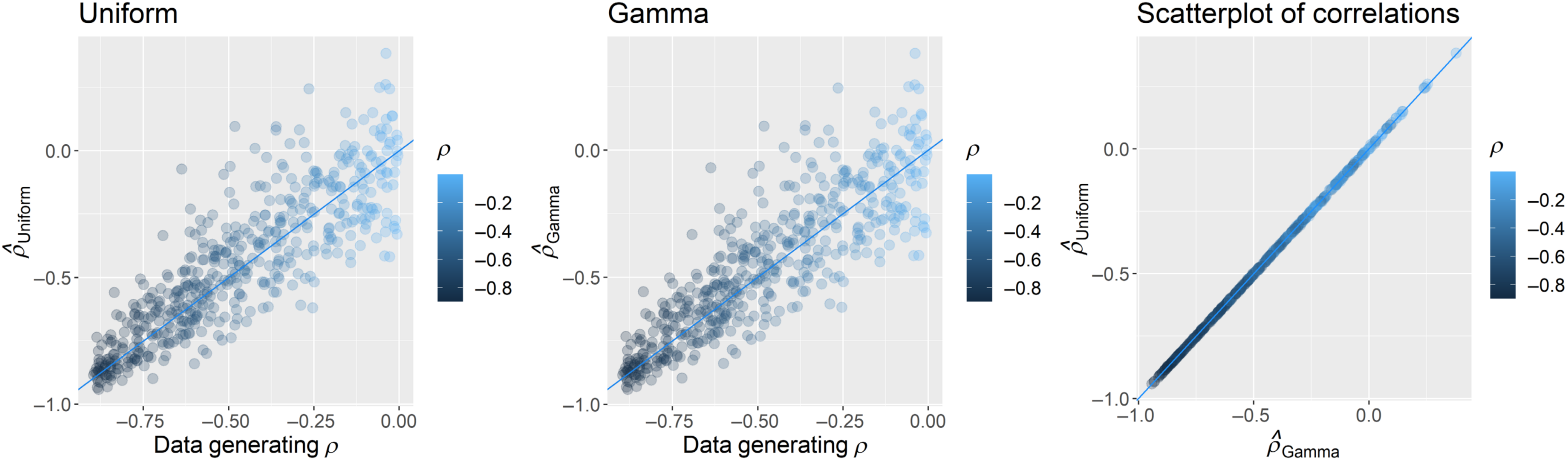
Scatterplots of the median estimated values of correlations between hunting and natural mortality hazard rates from models using uniform (left) and gamma (center) priors for mortality hazard rate standard deviations regressed against the data generating values of *ρ*, as well as the estimates from the two models regressed against each other (right). Note that these two models produce nearly identical estimates of correlations between mortality hazard rates (right), as there is little difference between the priors for standard deviations, no difference between the priors for correlations, and no dependency between the correlation and standard deviation parameters (Figure 2) for either model.

Both the ‘Uniform’ and ‘Gamma’ parameterizations we describe in Section 3 adequately recovered simulated correlation parameter values (Figure 1). Parameter coverage, or the proportion of the data generating parameter values that fall within the 95% credible interval of the correlation posterior distribution, was very slightly less than acceptable for both the gamma (.936) and uniform (.936) model parameterizations. The average distance between the median of the posterior distribution for the correlation parameter and the values used to generate simulated estimates were .023 for models using gamma priors, and .021 for uniform priors (Figure 1). If we compare parameter estimates to the actual simulated correlations (i.e., the correlations of the simulated points), coverage improved for both the gamma (.998) and uniform (.998) parameterizations, and the distance between the median of the posterior distributions and the true correlation of the simulated points was minimal for both gamma (−.0003) and uniform (−.002) models. In other words, the models accurately estimated the correlation of the observed data, but the correlation of the observed data may differ slightly from the true underlying correlation due to the duration of the study (*T* = 36). In summary, both approaches recovered the simulated parameter values and produced nearly identical results (Figure 1).

## 4 ERRORS IN INTERPRETATION AND INFERENCE

Deane et al. (2023) incorrectly interpreted the meaning of disparate results from their three priors. Specifically, they concluded that ‘using Gamma priors will lead to overestimating the magnitude of negative correlation and Wishart priors likely underestimated the magnitude of negative correlation between survival and recovery’. Later in the discussion, they concluded that ‘variable prior influence among different priors is an expected outcome of Bayesian estimation’.

This is not entirely correct. Our simulations reveal quite clearly that the Uniform and Gamma priors we describe here produced nearly identical estimates (Figure 1). When different vague priors are used with adequate data and implemented appropriately, we should expect posterior estimates to be robust. Critically, major changes in posterior distributions as a result of minor changes in prior distributions should serve as a warning sign for ecologists writing complex models with robust datasets. It is only when informative priors (e.g., the inverse Wishart prior), very ‘weak’ data, or simply incorrect equations (Deane et al., 2023) are used that we should expect major disparities in inference among priors.

**FIGURE 2:**
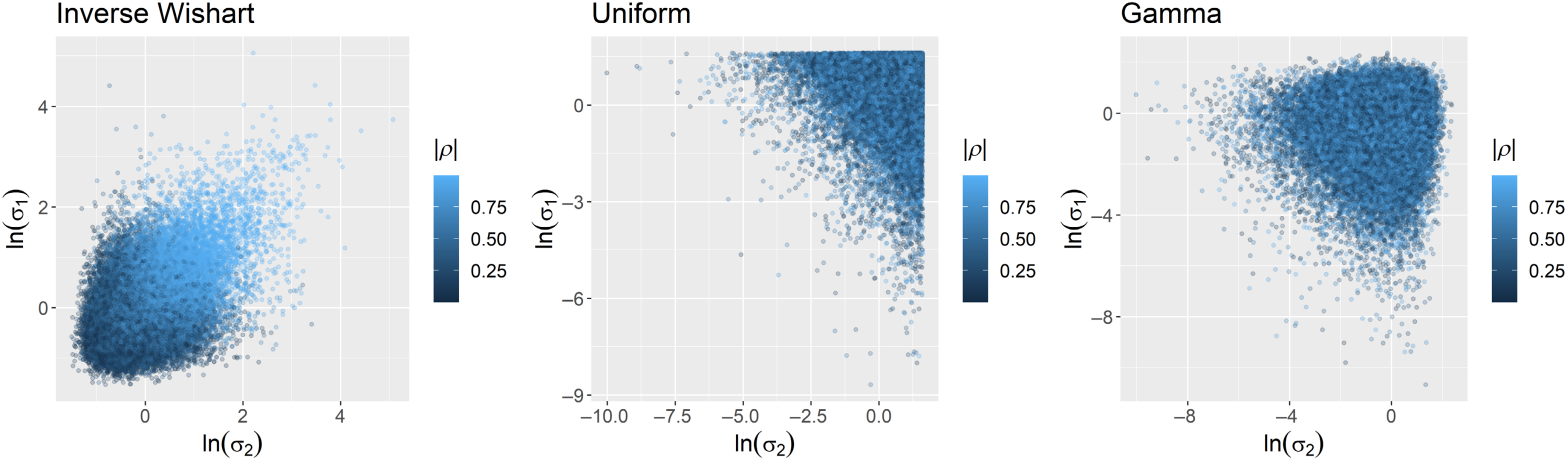
Scatterplots of the natural log of standard deviations (**σ**) drawn from inverse Wishart (left), Uniform (center), and Gamma (right) priors in which lighter blue colors indicate increased absolute values of the correlation parameter (*ρ*). Note that the inverse Wishart induces a dependency between the standard deviations and the correlation which can lead to under‐ or over‐estimation of the correlation as a function of the data and the prior (Alvarez et al., 2014; Riecke et al., 2019). The other two approaches do not induce the same dependency. In this example, this leads to accurate estimation of correlations among mortality hazard rates when using vague uniform or gamma priors for standard deviations of mortality hazard rates.

The conjugate inverse‐Wishart prior has been repeatedly shown to be problematic in these types of analyses (Alvarez et al., 2014; Fay et al., 2022; Link & Barker, 2005; Riecke et al., 2019). While its use often leads to underestimation of correlations in capture‐reencounter analyses due to an inherent dependency between the standard deviations and the correlation parameter(s), the shape of the logit‐link, and the ranges of typical demographic parameter estimates, this problem can cause either under‐ or overestimation of correlations (Figure 2). In Section 3, we demonstrated that uniform and gamma priors for standard deviations of mortality hazard rates resulted in nearly identical parameter estimates for correlations (Figure 1). Our results here and elsewhere (Riecke et al., 2019) suggest that these two approaches, which place vague priors on the standard deviations of the demographic parameters and do not induce dependencies between the standard deviations and the correlations (Figure 2), recover accurate estimates of simulated correlations when sample sizes are adequate (Riecke et al., 2019). As our simulations and basic theory indicate, the use of different vague priors in Bayesian analyses should not induce major variation in posterior estimates if the data are sufficient to estimate the parameters of interest. We suggest that Deane et al.'s (2023) conclusions are a consequence of misunderstandings of the equations and relevant literature underlying the simulations and analyses presented in their manuscript. We recommend that interested readers refer to other work (Alvarez et al., 2014; Ergon et al., 2018; Fay et al., 2022). Finally, we note that many expansions of these model types have been developed relatively recently, and that continued advances in Bayesian software development and statistics have led to additional useful parameterizations.

## 5 CONCLUSION

The continued expansion of Bayesian software and the careful and thoughtful work of statisticians has led to a plethora of options for placing priors on correlated random effects drawn from multivariate normal distributions. These include but are not limited to; scaled inverse Wishart distributions (O'Malley & Zaslavsky, 2008), hierarchical half‐t priors (Huang & Wand, 2013), the separation strategy proposed by Barnard et al. (2000), Lewandoski‐Kurowicka‐Joe priors (Lewandowski et al., 2009) as well as various modifications of the Cholesky decomposition approach (Chen & Dunson, 2003; Dunson, 2008; Fay et al., 2022). When using bivariate normal distributions, researchers can simply assign hyperpriors to each component of the covariance matrix (Riecke et al., 2019). While the approaches that involve inverse Wishart distributions (Huang & Wand, 2013; O'Malley & Zaslavsky, 2008) retain some of the same challenges of the inverse Wishart, all of the approaches listed above have advantages over the conjugate inverse Wishart prior. Further, they can be implemented using a wide array of Bayesian software such as JAGS (Plummer, 2003), NIMBLE (de Valpine et al., 2017), or Stan (Gelman et al., 2015), and many are readily extendable to covariance matrices larger than 2 × 2. We note that these approaches are complex (Alvarez et al., 2014; Tokuda et al., 2011) and we strongly recommend that ecologists and wildlife managers interested in exploring these concepts use one of the many well‐documented existing approaches developed by formally trained statisticians.

Although we have been critical in our letter, we do find areas of agreement with Deane et al. (2023). Like other related manuscripts (Fay et al., 2022; Riecke et al., 2019), Deane et al. (2023) demonstrated challenges associated with use of the inverse Wishart prior when estimating correlations among parameters. Similar to other recent work (Fay et al., 2022; Riecke et al., 2019), Deane et al. (2023) demonstrated a need for assessing variation in the annual number of marked individuals for estimating correlations. We note that study duration may also be an important metric to consider when assessing posterior distributions of correlations, and that simply estimating correlations without considering underlying causal processes may lead to flawed inference (Riecke, Lohman, et al., 2022; Riecke, Sedinger, et al., 2022). Perhaps most importantly, we agree with Deane et al. (2023) that power analyses, or simulation‐based model validation, are an important component of assessing quality of inference. Deane et al. (2023) wrote that, ‘studies should demonstrate through simulation or power analysis that the ecological questions being assessed can be answered with the data and statistical methods employed’ We fully agree and add that when complex problems are addressed, practitioners should take additional care to check their equations and code to ensure that the model parameterizations they have chosen are sensible, and that equations are accurate.

We again acknowledge that these techniques are complicated. As such, we strongly recommend that researchers that are unfamiliar with these approaches consult the literature and confer with one or more statisticians if possible as they implement these model types. Failure to do so can lead to inappropriate inference. As a final note, we strongly encourage data and code sharing as part of the publication process (Jenkins et al., 2023). Much of our response would not have been possible if the code from Deane et al. (2023) was not publicly available.

## AUTHOR CONTRIBUTIONS

**Thomas V. Riecke:** Conceptualization (lead); methodology (equal); writing – original draft (lead); writing – review and editing (equal). **Dan Gibson:** Conceptualization (equal); methodology (equal); writing – review and editing (equal). **James S. Sedinger:** Conceptualization (equal); methodology (equal); writing – review and editing (equal).

## CONFLICT OF INTEREST STATEMENT

We declare no conflict of interest.

## FUNDING INFORMATION

No funding contributed to this work.

## Data Availability

The R script used to simulate and analyze data and create figures as well as the Stan model files are available from the Dryad Digital Repository (https://datadryad.org/stash/dataset/doi.org/10.5061/dryad.3bk3j9kv6; Riecke et al., 2024).

## References

[ece370286-bib-0001] Alvarez, I. , Niemi, J. , & Simpson, M. (2014). Bayesian inference for a covariance matrix . arXiv preprint arXic:1408.4050.

[ece370286-bib-0025] Barnard, J. , McCulloch, R. , & Meng, X. L. (2000). Modeling covariance matrices in terms of standard deviations and correlations, with application to shrinkage. Statistica Sinica, 10, 1281–1311.

[ece370286-bib-0002] Brownie, C. , Anderson, D. R. , Burnham, K. P. , & Robson, D. S. (1985). Statistical inference from band recovery data: A handbook (Vol. 156). U. S. Fish and Wildlife Service Resource Publication.

[ece370286-bib-0003] Chen, Z. , & Dunson, D. B. (2003). Random effects selection in linear mixed models. Biometrics, 59, 762–769. 14969453 10.1111/j.0006-341x.2003.00089.x

[ece370286-bib-0004] de Valpine, P. , Turek, D. , Paciorek, C. J. , Anderson‐Bergman, C. , Lang, D. T. , & Bodik, R. (2017). Programming with models: Writing statistical algorithms for general model structures with NIMBLE. Journal of Computational and Graphical Statistics, 26(2), 403–413.

[ece370286-bib-0005] Deane, C. E. , Carlson, L. G. , Cunningham, C. J. , Doak, P. , Kielland, K. , & Breed, G. A. (2023). Prior choice and data requirements of Bayesian multivariate hierarchical models fit to tag‐recovery data: The need for power analyses. Ecology and Evolution, 13, e9847. 36993148 10.1002/ece3.9847 PMC10041078

[ece370286-bib-0006] Dunson, D. B. (2008). Random effect and latent variable model selection. Springer.

[ece370286-bib-0007] Ergon, T. , Borgan, O. , Nater, C. R. , & Vindenes, Y. (2018). The utility of mortality hazard rates in population analyses. Methods in Ecology and Evolution, 9, 2046–2056.

[ece370286-bib-0008] Fay, R. , Authier, M. , Hamel, S. , Jenouvrier, S. , van de Pol, M. , Cam, E. , Gaillard, J.‐M. , Yoccoz, N. G. , Acker, P. , Allen, A. , Aubry, L. M. , Bonenfant, C. , Caswell, H. , Coste, C. F. D. , Larue, B. , Le Couer, C. , Gamelon, M. , Macdonald, K. R. , Moiron, M. , … Sæther, B.‐E. (2022). Quantifying fixed individual heterogeneity in demographic parameters: Performance of correlated random effects for Bernoulli variables. Methods in Ecology and Evolution, 13, 91–104.

[ece370286-bib-0009] Gelman, A. , Lee, D. , & Guo, J. (2015). Stan: A probabilistic programming language for Bayesian inference and optimization. Journal of Educational and Behavioral Statistics, 40, 530–543.

[ece370286-bib-0028] Huang, A. , & Wand, M. P. (2013). Simple marginally noninformative prior distributions for covariance matrices. Bayesian Analysis, 8, 439–452. 10.1214/13-­BA815

[ece370286-bib-0010] Jenkins, G. B. , Beckerman, A. P. , Bellard, C. , Benítez‐López, A. , Ellison, A. M. , Foote, C. G. , Hufton, A. L. , Lashley, M. A. , Lortie, C. J. , Ma, Z. , Moore, A. J. , Narum, S. R. , Nilsson, J. , O'Boyle, B. , Provete, D. B. , Razgour, O. , Rieseberg, L. , Riginos, C. , Santini, L. , … Peres‐Neto, P. R. (2023). Reproducibility in ecology and evolution: Minimum standards for data and code. Ecology and Evolution, 13, e9961. 37181203 10.1002/ece3.9961 PMC10170304

[ece370286-bib-0011] Lauritzen, S. L. (1996). Graphical models. Oxford University Press.

[ece370286-bib-0012] Lewandowski, D. , Kurowicka, D. , & Joe, H. (2009). Generating random correlation matrices based on vines and extended onion method. Journal of Multivariate Analysis, 100, 1989–2001.

[ece370286-bib-0013] Link, W. A. , & Barker, R. J. (2005). Modeling associations among demographic parameters in analysis of open population capture‐recapture data. Biometrics, 61, 46–54. 15737077 10.1111/j.0006-341X.2005.030906.x

[ece370286-bib-0027] O'Malley, A. J. , & Zaslavsky, A. M. (2008). Domain‐level covariance analysis for multilevel survey data with structured nonresponse. Journal of the American Statistical Association, 103, 1405–1418. 10.1198/016214508000000724

[ece370286-bib-0014] Plummer, M. (2003). JAGS: A program for analysis of Bayesian graphical models using Gibbs sampling. In Proceedings of the 3rd international workshop on distributed statistical computing (p. 125). Technische Universit at Wien.

[ece370286-bib-0026] R Core Team (2023). R: a language and environment for statistical computing. R Foundation for Statistical Computing, Vienna, Austria. https://www.R‐project.org

[ece370286-bib-0015] Riecke, T. V. , Gibson, D. , & Sedinger, J. S. (2024). Data from: Accurately estimating correlations between demographic parameters: A comment on Deane et al. (2023). 10.5061/dryad.3bk3j9kv6 PMC11408121 39296737

[ece370286-bib-0016] Riecke, T. V. , Lohman, M. G. , Sedinger, B. S. , Arnold, T. W. , Feldheim, C. L. , Koons, D. N. , Rohwer, F. C. , Schaub, M. , Williams, P. J. , & Sedinger, J. S. (2022). Density‐dependence produces spurious relationships among demographic parameters in a harvested species. Journal of Animal Ecology, 91, 2261–2272. 36054772 10.1111/1365-2656.13807 PMC9826280

[ece370286-bib-0017] Riecke, T. V. , Sedinger, B. S. , Arnold, T. W. , Gibson, D. , Koons, D. N. , Lohman, M. G. , Schaub, M. , Williams, P. J. , & Sedinger, J. S. (2022). A hierarchical model for jointly assessing ecological and anthropogenic impacts on animal demography. Journal of Animal Ecology, 91, 1612–1626. 35603988 10.1111/1365-2656.13747 PMC9543922

[ece370286-bib-0018] Riecke, T. V. , Sedinger, B. S. , Williams, P. J. , Leach, A. G. , & Sedinger, J. S. (2019). Estimating correlations among demographic parameters in population models. Ecology and Evolution, 9, 13521–13531. 31871663 10.1002/ece3.5809 PMC6912887

[ece370286-bib-0019] Stan Development Team . (2024a). Stan modeling language users guide and reference manual . https://mc‐stan.org/

[ece370286-bib-0020] Stan Development Team . (2024b). RStan: The R interface to Stan . R package version 2.32.6. https://mc‐stan.org/

[ece370286-bib-0022] Tokuda, T. , Goodrich, B. , van Mechelen, I. , Gelman, A. , & Tuerlinckx, F. (2011). *Visualizing distributions of covariance matrices* [Unpublished report]. http://stat.columbia.edu/~gelman/research/unpublished/Visualization.pdf

[ece370286-bib-0024] White, G. C. , & Burnham, K. P. (1999). Program MARK: survival estimation from populations of marked animals. Bird Study, 46, S120– S139.

[ece370286-bib-0023] Zipkin, E. F. , Doser, J. W. , Davis, C. L. , Leuenberger, W. , Ayebare, S. , & Davis, K. L. (2023). Integrated community models: A framework combining multispecies data sources to estimate the status, trends and dynamics of biodiversity. Journal of Animal Ecology, 92, 2248–2262. 37880838 10.1111/1365-2656.14012

